# Simulation-based inference for subject-specific tuning of middle ear finite-element models towards personalized objective diagnosis

**DOI:** 10.1038/s41598-025-22164-2

**Published:** 2025-11-03

**Authors:** Hamid Motallebzadeh, Michael Deistler, Florian M. Schönleitner, Jakob H. Macke, Sunil Puria

**Affiliations:** 1 Department of Communication Sciences and Disorders, California State University, Sacramento, CA USA; 2 Department of BioMedical Engineering, McGill University, Montreal, QC Canada; 3 Machine Learning in Science, University of Tübingen, Tübingen, Germany; 4 Tübingen AI Center, Tübingen, Germany; 5 TUM School of Computation, Information and Technology, Garching, Germany; 6 Department Empirical Inference, Max Planck Institute for Intelligent Systems, Tübingen, Germany; 7 Eaton-Peabody Laboratories, Massachusetts Eye and Ear, Boston, USA; 8 Speech and Hearing Bioscience and Technology, Harvard University Graduate School of Arts and Sciences, Cambridge, USA

**Keywords:** Simulation-based inference, Finite-element method, Tuning mechanistic models, Patient-specific models, Computational models, Translational research, Biomedical engineering

## Abstract

Computational models, particularly finite-element (FE) models, are essential for interpreting experimental data and predicting system behavior, especially when direct measurements are limited. Tuning these models is particularly challenging when a large number of parameters are involved. Traditional methods, such as sensitivity analyses, are time-consuming and often provide only a single set of parameter values, focusing on reproducing averaged trends rather than capturing experimental variability. New approaches are needed to make computational models more adaptable to patient-specific clinical applications. We applied simulation-based inference (SBI) using neural posterior estimation (NPE) to tune an FE model of the human middle ear against subject-specific data. The training dataset consisted of 10,000 FE simulations of stapes velocity, ear-canal input impedance, and absorbance, paired with seven FE parameter values sampled within plausible ranges. By using simulated data, we generated a diverse training dataset, enabling efficient learning by the neural network (NN). The NN learned the association between parameters and simulation outcomes, providing a probability distribution of parameter values, which could be used to produce subject-specific computational inferences. By accounting for noise and test–retest variability, the method provided a probability distribution of parameters, rather than a single set, fitting three experimental datasets simultaneously. Importantly, examining the inferred parameter distributions alongside prior knowledge of normal ranges enables individualized differential inference used for diagnosis. SBI offers an objective alternative to sensitivity analyses, uncovering parameter interactions, supporting personalized diagnosis and treatment, and compensating for limited clinical training data. This method is applicable to any computational model, enhancing its potential for improved patient outcomes.

**Supplementary Information:**

The online version contains supplementary material available at 10.1038/s41598-025-22164-2.

## Introduction

Computational models are essential tools for explaining experimental data and predicting system behavior, particularly when direct measurements are challenging due to practical limitations^1^. In clinical diagnostics, computational models facilitate non-invasive predictions and support personalized healthcare, offering new opportunities for precision medicine. These models have recently been employed to generate synthetic data for training machine learning algorithms, which help reduce the need for large datasets of clinically confirmed pathological cases that are often difficult to access due to their scarcity and limited representation in available databases^2^. This capability alleviates data scarcity, a persistent challenge in healthcare, by enabling model-based data generation. Moreover, computational models hold significant promise for developing patient-specific models that can objectively interpret diagnostic tests and assist in designing personalized intervention plans.

Among the variety of computational models, finite-element (FE) models are particularly advantageous for representing the physical and geometrical details of biological systems with high fidelity, typically reconstructed from imaging methods such as CT or micro-CT^3^. However, developing valid FE models presents considerable challenges, primarily due to the large number of parameters that need to be specified. Key challenges in FE modeling include assigning material properties, defining boundary conditions, and making justified simplifications.

Assigning material properties is difficult because a wide range of these values is reported in the literature. For instance, the Young’s modulus of the eardrum has been reported to range from 2 to 300 MPa, depending on the experimental setup and treatment of the eardrum sublayers^4,5^. When direct measurements are unavailable, parameters must often be adopted from similar tissues^6^. Additionally, defining material models for FE simulations involves complexities such as anisotropy^7^, viscoelasticity, and nonlinearities^8,9^. The selection of material models often depends on the specific objectives of the simulation, computational resources, and parameter availability.

Another significant challenge is the assignment of boundary conditions and the need for justified simplifications. For example, simplifying the eardrum extension by clamping its annular ligament affects its mechanical behavior^10^. Similarly, modeling the middle ear (ME) cavity as a smooth, lossless hard wall oversimplifies its complex air-cell structure^11,12^. These simplifications can impact model fidelity and the interpretation of simulation results.

Ensuring that the data extraction from FE simulations mirrors experimental setups is critical for model validation and accurate interpretation of results. For example, experimental vibrometry typically uses narrow tube microphones placed near the umbo to measure pressure, while FE models often average pressure over the entire ear canal^13,14^. Additionally, uncertainties in the measurement direction arise due to experimental limitations, leading to non-perpendicular trajectories, which can accentuate certain vibration modes and affect accuracy^15,16^. These differences between experimental and simulated measurement setups highlight the importance of aligning the two to achieve model validation.

Conventionally, tuning FE models involves one-by-one parameter sensitivity analyses, iteratively comparing simulation results with experimental measurements^10^. This process is time-intensive, highly subjective, and often overlooks complex interactions between parameters^17,18^. Furthermore, experimental data is often represented as a population average, masking individual variability. As a result, the development and refinement of a comprehensive ME model can take several years. For example, one of our ME FE models^10^ required 6–7 years of refinement, starting with earlier work^19^ and progressing through multiple iterations^20^.

There have been attempts at semi-automatic optimization of FE models^9,21^. However, these methods have been limited, often focusing on one or two parameters simultaneously. Sackmann et al.^22^ introduced inverse fuzzy arithmetic for parameter identification in the ME to detect pathological conditions, providing a possibilistic assessment based on imprecise or incomplete data. In a follow-up study, Sackmann et al.^23^ selected multiple fitting criteria, such as resonance characteristics, and used averaged sensitivity indices to identify parameter values for a simplified ME model based on reference experimental data^24^. These methods have also been used to classify ME pathologies^2^. However, machine-learning algorithms trained on discrete simulators may misinterpret normal intersubject variability in ME structures as pathological conditions^25^.

To address these limitations, we applied simulation-based inference (SBI) using Neural Posterior Estimation (NPE)^26,27^ to tune an FE model of the human ME against subject-specific data. Unlike traditional approaches that rely on population-averaged data, our method aims to capture individual variability by inferring the posterior distribution of parameters from experimental data^28^.

In NPE, parameter sets are initially sampled from a prior distribution within plausible ranges and used to generate simulated input–output pairs for training a deep neural density estimator^29^. This approach leverages the knowledge embedded in validated FE models, making it more practical than extensive physiological or clinical measurements, where input–output relationships may not be well understood. NPE provides a principled way to handle noisy measurements using Bayesian inference to determine parameter settings consistent with the data, and it can be amortized, meaning once trained, it can rapidly infer parameters from new data without requiring additional simulations.

By leveraging SBI, our approach offers an objective alternative to conventional sensitivity analyses for parameter identification, supporting individualized tuning of FE models that can then be used for improving the accuracy of patient-specific diagnostics. This advancement holds transformative potential for computational audiology, particularly in personalized diagnostics and intervention planning, ultimately improving patient outcomes through more accurate and efficient care. By enabling the development of personalized FE models, clinicians can achieve faster, more accurate diagnoses, offering a new frontier for precision healthcare.

## Results

**Fig. 1:**
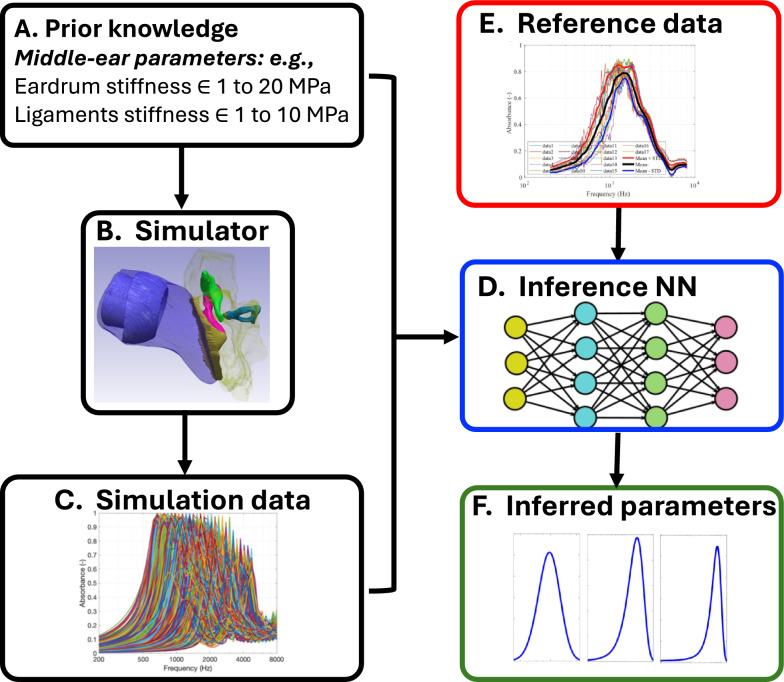
Schematic flow of SBI: (**A**) Prior range of simulator parameters are identified, and a subset of random values within this range selected and (**B**) imported into a finite element model termed mechanistic ‘simulator’ to generate the (**C**) simulation data. Multiple simulations are performed. (**D**) The paired sets of simulator parameter inputs and simulation data outputs are then introduced to the SBI neural network (NN), which learns their input–output relationships and that effectively trains a NN. (**E**, **F**) When a reference data set (measurements) is introduced, the NN infers the probability distributions of the parameters that would have produced that data.

**Fig. 2:**
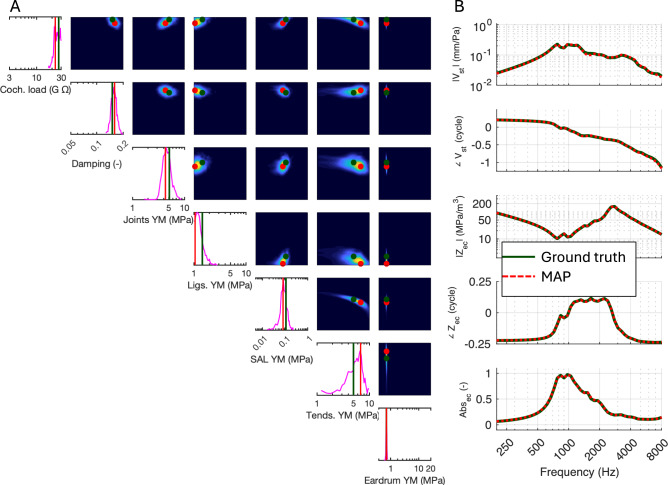
SBI Validation. The SBI baseline NN was trained with 10,000 simulations. A new set of random parameter values (dark green vertical lines and dots in **A**) was input into the FE simulator. The resulting stapes velocity (V_st_/P_ec_), impedance (Z_ec_), and absorbance (Abs_ec_) at 50 frequencies (dark green lines in **B**) were fed into the trained NN. The NN returned 1D probability distributions for each parameter (magenta histogram lines in the diagonal panels of **A**) and paired histograms as confusion matrices (off-diagonal color maps in **A**). The NN also returned the MAP values for each parameter (red vertical lines and dots in **A**). These MAP values were imported into the FE simulator, and the resulting stapes velocity, impedance, and absorbance are plotted as red dotted lines in **B**, overlapping with the original data (black lines). Dots in **A** represent parameter samples projected onto the 1D and 2D histograms (green for input sample, red for maximum *a posteriori* (MAP) estimate), and their corresponding model responses are plotted in the same color in panel **B**.

**Fig. 3:**
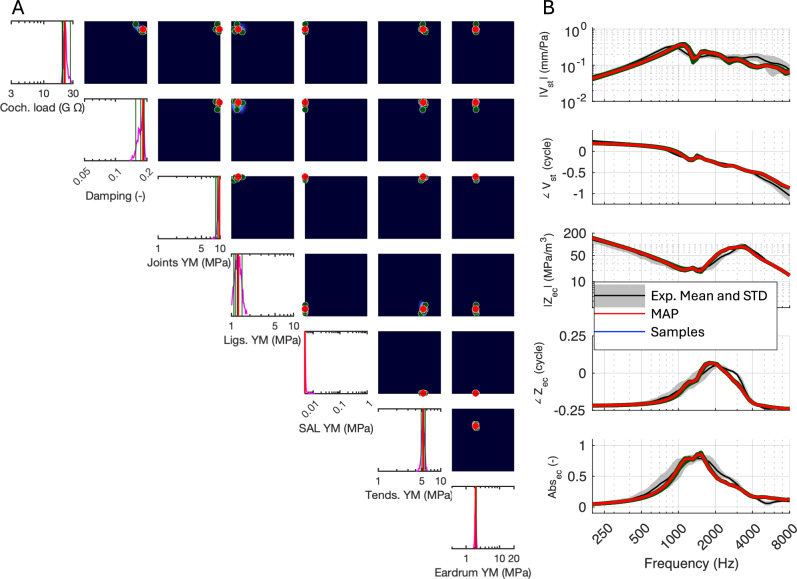
Inferring the model parameters from reference experimental data. Reference data averaged over 17 test–retest measurements from a single temporal bone was used to determine parameters from the initially trained NN (Fig. 2). (**A**) For the given input, the NN returned the MAP (red vertical lines and dots) and five arbitrarily chosen parameter sets (dark-green vertical lines and dots) within the parameter probability distributions (magenta curves), which were then imported into the FE simulator. (**B**) The resultant spectra (red line) for the MAP and the five samples (dark-green lines) are compared with the reference experimental data (black curves). Dots in **A** correspond to the parameter sets (MAP in red, samples in green), and the resulting model responses are shown in matching colors in **B**. The MAP-generated spectra largely overlap with the green sample curves, indicating low variability and strong agreement. As a result, the individual green curves are not fully visible in panel **B**.

**Fig. 4:**
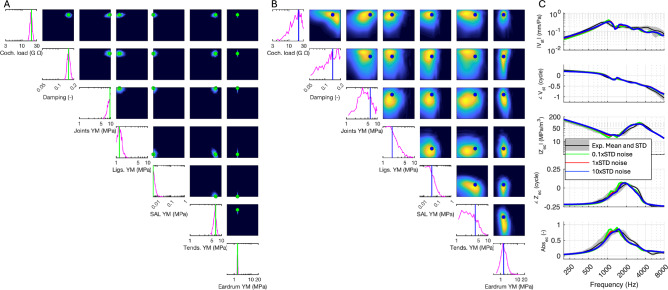
The effect of added noise to the training data on NN performance**:** Baseline noise (1 × STD) was extracted from experimental data and added to simulation data from the FE simulator, then scaled by factors of 0.1, 1, and 10. These modified datasets were used to train the NN. The three trained NNs were then tested with the experimental data, returning parameter probability distributions for (**A**) 0.1xSTD, 1xSTD, and (**B**) 10xSTD. Descriptions of figures and NN output for 1xSTD noise are shown in Fig. 3. MAP estimates are indicated by green dots (**A**: 0.1 × STD), red dots (1 × STD, shown in Fig. 3), and blue dots (**B**: 10 × STD). (**C**) The MAP values of the predicted distributions were imported into the FE model, and the results were compared with experimental data. Model responses corresponding to MAP values are shown in matching colors (green, red, and blue).

**Fig. 5:**
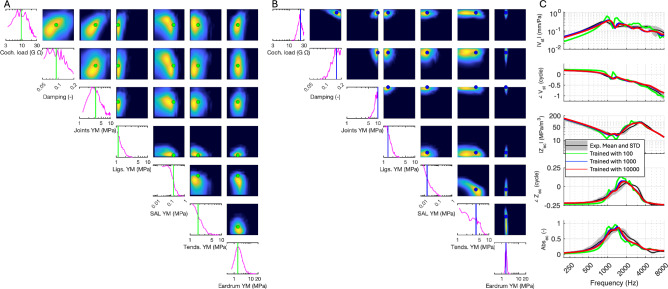
Effect of training dataset size on NN performance**:** The NN was trained with 100, 1000, and 10,000 datasets. The three trained NNs were then exposed to the experimental data, returning the probability distributions of the parameters for (**A**) 100 and (**B**) 1000 FE model simulations. The results for 10,000 model simulations and the panel descriptions are presented in Fig. 3. (**C**) The MAP values of the predicted distributions were imported into the simulator, and the resultant spectra were compared with the experimental data.

A schematic overview of the SBI methodology is shown in Fig. 1 and the pipeline for the simulation-based inference (SBI) methodology is outlined in the Methods section. We first evaluated the performance of the trained SBI model on FE simulation data generated using ground truth known parameters (Fig. 2). Next, we applied the trained NN to infer the parameters of the baseline FE model using reference experimental data (Fig. 3). To assess the robustness of the SBI, we performed a sensitivity analysis on varying additive noise levels (Fig. 4). The effect of the training dataset size was also assessed (Fig. 5). The effects of data extraction location and training the NN with individual reference data are detailed in the supporting information.

### Neural network training and validation

We randomly sampled parameter values within the prior ranges (Table S1) and performed 10,000 simulations, each pairing the sampled parameter sets with corresponding results, including normalized stapes velocity (V_st_/P_ec_, where V_st_ is the stapes velocity and P_ec_ is the ear canal pressure), ear canal impedance (Z_ec_), and absorbance (Abs_ec_) at 50 logarithmically spaced frequencies from 200 to 8000 Hz. These features were chosen to capture the key biomechanical and acoustic responses of the ME system. Using this dataset, we trained the baseline NN.

We then generated five sets of previously unseen parameters for the FE simulator and obtained the corresponding simulation results. These test sets allow for an independent evaluation of model generalization. One of these sets is shown as an example in Fig. 2B (dark green curves). These spectra were input into the trained NN, which returned probability distributions for each parameter based on the inferred posterior distributions (magenta lines) and maximum *a posteriori* (MAP) values (red dots and vertical lines, Fig. 2A). The diagonal panels of Fig. 2A display 1D marginal distributions, while the upper diagonal panels show 2D paired distributions, highlighting interdependencies and correlations between parameter pairs. Key findings from the analysis include the following:

1. MAP estimates were consistently close to the true parameter values, with all MAP estimates falling within the predicted posterior distributions.
2. Parameters such as the Young’s modulus of the eardrum and damping exhibited narrow posterior distributions, indicating high sensitivity to these parameters.
3. Parameters such as the Young’s moduli of tendons showed broader distributions, suggesting lower sensitivity for these parameters.

The pairwise marginal distributions (Fig. 2A, color maps) reveal parameter interactions in multi-dimensional space. MAP values (red dots) do not always align with the marginal peaks, reflecting the influence of higher-order correlations captured by SBI. These off-peak MAP locations underscore the ability of SBI to account for complex interactions that may not be visible in one-dimensional projections.

Finally, we used the predicted MAP values as input to the FE simulator and compared the resulting output spectra (dashed red curves) with the ground truth spectra (solid dark green curves) (Fig. 2B). This comparison directly assesses the accuracy of the parameter estimates produced by the trained NN. In all cases, the predicted spectra showed a close match to the ground truth, demonstrating the accuracy of the model input parameters predicted by the NN. Notably, the spectral match was consistent across the entire frequency range of 200 to 8000 Hz, indicating that the model can generalize well across diverse frequency-dependent behaviors.

As a naïve measure of similarity, we calculated Spearman’s rank correlation coefficient (ρ) between the MAP-predicted spectra and the ground truth. This non-parametric metric assesses the monotonic relationship between two variables based on rank ordering. High correlations were observed for stapes velocity magnitude (ρ = 0.995), stapes velocity phase (ρ = 0.999), impedance magnitude (ρ = 1.000), impedance phase (ρ = 0.999), and absorbance (ρ = 0.999), with a mean correlation of ρ = 0.998 across all features.

### Inferring the ME parameters of the experimental dataset

After validating the trained SBI, we imported the mean spectral data from experimental measurements into the trained NN to infer model parameters that could reproduce similar responses (Fig. 3). The experimental data consisted of test–retest measurements repeated 17 times on the same sample, allowing for an accurate characterization of measurement variability (see Methods for more details). The SBI produced probability distributions (magenta curves) and MAP estimates (red vertical lines and dots), as shown in Fig. 3A. This step aimed to identify the parameter set that best explains the observed experimental responses. The variability in the experimental data, represented by standard deviations across all experiments, was incorporated into the NN training as plausible noise to ensure robustness to experimental uncertainty (Methods: Training the inference neural network).

Although the mean of the experimental dataset was used to infer parameter values, SBI incorporates the variability present in test–retest data during training. Since the FE simulator is a deterministic model, it does not inherently account for experimental noise. To address this, we introduced plausible random noise to the simulated data before feeding it into the NN during training (step C to D in Fig. 1). The magnitude of this noise was based on the observed standard deviations (STD) in the experimental test–retest data (gray areas in Fig. 3B). This approach ensures that the SBI training process reflects real-world variability, enabling the model to generalize better to experimental data.

The results in Fig. 3A indicate that the Young’s moduli of the ligaments (row 4) and stapedial annular ligament (row 5) were biased toward the lower limits of their prior ranges, while the Young’s modulus of joints (row 3) converged at the upper boundary. The Young’s modulus of the eardrum (row 6) exhibited a distinct peak centered around 3 MPa, consistent with the lower range of previous studies (see Introduction). This convergence around 3 MPa aligns with known estimates from previous experimental work, supporting the biological plausibility of the inferred values.

To demonstrate the robustness of the inferred values, we sampled five sets of parameter values from the inferred probability distribution and performed FE simulations with these values (Fig. 3A, dark green vertical lines in 1D and points in 2D paired histograms). This approach provided insight into the variability of potential parameter sets that produce similar spectral responses. The simulated spectral data from both the MAP estimates and the five samples (Fig. 3B, red and dark green curves, respectively) aligned closely with the experimental data (black lines with gray standard deviations). The close agreement across the entire frequency range supports the reliability of the inferred parameter distributions. To quantify this agreement, we calculated the Spearman rank correlation (ρ) between the MAP-simulated spectra and the experimental means: ρ = 0.916 for |Vst|, 0.999 for ∠Vst, 0.975 for |Zec|, 0.992 for ∠Zec, and 0.937 for Abs_ec_, with an overall mean |ρ| of 0.964.

We also explored the impact of training the SBI on individual datasets—stapes velocity (V_st_/P_ec_), impedance (Z_ec_), and absorbance magnitude (Abs_ec_)—compared to using all three datasets together. To investigate this, we trained new NNs for each individual dataset separately and compared their performance to NNs trained on the combined datasets (Fig. SI-1). Previous studies (6) often used only ear impedance to estimate FE parameters. We sought to evaluate if incorporating multiple datasets (V_st_/P_ec_, Z_ec_, and Abs_ec_) would improve the predictive capacity of SBI. As shown in Fig. SI-1, when trained on individual reference experimental sets (Fig. SI-1A to C), the FE model was unable to accurately reproduce the other experimental sets. This result highlights the limitations of training with only a single experimental dataset. However, when trained on all three datasets simultaneously, it was able to adequately reproduce all reference datasets, demonstrating the enhanced predictive power of SBI when incorporating multi-modal experimental data (Fig. SI-1D). This finding underscores the value of multi-feature training for improving the generalizability and robustness of the parameter inference process, highlighting that an FE model tuned to reproduce one experimental feature may not be adequately tuned to capture other features.

### Data noise and robustness of SBI

To account for measurement noise and intrasubject variability, Gaussian noise was added to the simulations used to train the NN, as explained in the previous section. We assessed SBI’s robustness across different noise scales, ranging from 0.1 × to 10 × the experimentally measured STD of velocity, impedance, and absorbance data. This approach allowed us to evaluate the impact of noise on parameter distributions and model robustness. NNs were trained using varying noise levels applied to the simulator output following the process illustrated in Fig. 1C.

Experimental data were then input into the trained NNs, generating individual and paired histograms of parameter distributions (Fig. 4 A and B). The MAP parameter values were fed back into the FE simulator to produce spectra for comparison with the experimental reference data (Fig. 4C). As expected, increasing the noise to 10xSTD broadened the parameter distributions, while reducing noise to 0.1xSTD narrowed them (Fig. 4B vs. Figure 4A). This trend is consistent with the expected behavior of Bayesian inference, where higher uncertainty in the data leads to more diffuse posterior distributions. Despite the variations in noise, the MAP parameter values consistently reproduced the experimental data (Fig. 4C), demonstrating the NN’s robustness across different noise conditions, though larger noise levels led to broader distribution predictions. This indicates that the SBI approach can maintain accurate predictions even under substantial data variability. The results for the 1xSTD noise level (Fig. 4C, red curves) were identical to the baseline shown in Fig. 3.

These findings highlight the robustness of multi-feature training for handling noisy data and demonstrate that an FE model tuned to reproduce one experimental feature may not be adequately tuned to extract other features. By incorporating variability from multiple experimental features (velocity, impedance, and absorbance), the SBI approach achieves a more comprehensive and generalizable parameter inference process. This broader generalizability ensures that models trained with multi-feature data are less susceptible to overfitting to one specific feature or experimental noise.

### Effect of training dataset size

As outlined in the Methods section, we used 10,000 simulations to train the baseline NN within the SBI framework. To evaluate the effect of reducing the training dataset size, NNs were trained using 100 and 1000 datasets (Fig. 5). This generated individual and paired histograms of parameter distributions, and MAP parameter values were fed into the FE simulator to generate spectra for comparison with experimental data.

When trained with 100 datasets, the NNs produced broader posterior distributions (Fig. 5A), reflecting higher uncertainty in parameter estimates. This effect is most pronounced for parameters with lower sensitivity, such as Young’s moduli of tendons, where broader distributions are observed. Increasing the training size to 1000 datasets (Fig. 5B) significantly reduced this uncertainty, yielding narrower posterior distributions and more precise MAP estimates that were closer to the ground truth values. The posterior distributions of the NN (baseline) trained by 10,000 datasets is shown in Fig. 3.

The FE spectra corresponding to the MAP values are shown in Fig. 5C, where results from the 100-, 1000-, and 10,000-simulation NNs are compared with the experimental reference. The spectra generated from the 100-simulation NN exhibited larger deviations, particularly at higher frequencies. The spectra for the 1000- and 10,000-dataset NNs were closely aligned with the experimental reference, indicating that increasing the training size beyond 1000 provides only marginal improvement in spectral alignment. However, the narrower parameter distributions at 10,000 simulations suggest greater confidence in parameter estimates, as seen in Fig. 5B.

These findings illustrate how training dataset size influences parameter inference and spectral alignment. While using 1000 training samples achieves sufficient spectral alignment, larger training datasets (e.g., 10,000) produce narrower posterior distributions, reflecting increased confidence in the inferred parameter values. This trade-off between computational efficiency and certainty in parameter estimates is a key consideration in applications where generating large simulation datasets is computationally expensive.

## Discussion

In this study, we applied SBI to tune a finite-element model of a human ME simultaneously with multiple sets of experimental measurements. This approach represents a shift from traditional methods, such as sensitivity analysis, which are time-consuming, labor-intensive, and limited by their focus on one parameter at a time. A major advantage of SBI is its ability to provide probability distributions for parameter values and their interactions, offering quantitative insights into the sensitivity of model parameters to measured data. Moreover, while input data noise increased the variance of the parameter distributions, it had minimal impact on the MAP values and the overall simulation results.

Historically, manual refinement of a single, anatomically similar FE model of the ME in our group has taken 6–7 years of incremental sensitivity analyses and parameter tuning. In contrast, the entire SBI training set—comprising 10,000 simulations across 50 frequencies—was generated in just four weeks. However, the most important advantage lies in the trained neural network, which enables inference of patient-specific parameter estimates in under one minute. This reduces the end-to-end modeling timeline from years to weeks, provided that the model is appropriately parameterized for both material properties and anatomical geometry.

### Model validation and tuning

We trained the SBI NN with synthetic data, and then introduced FE simulation results from known model parameters. The NN returned predicted parameter distributions and MAP values that closely matched the ground truth (Fig. 2). Once the NN’s accuracy was confirmed, we introduced experimental data consisting of the mean of three test–retest measurements conducted on the same specimen, which included inherent experimental variability (not intersubject differences). The NN returned MAP values that, when imported into the FE model, generated responses closely aligned with the reference experimental data (Fig. 3).

Quantifying the similarity between predicted and experimental spectra is non-trivial. The experimental data represent means with standard deviations across repeated measurements, while the NN outputs a full set of posterior parameters, from which multiple spectra can be simulated. Conventional scalar metrics such as RMSE or direct pointwise errors are ill-suited across heterogeneous features like absorbance, impedance and velocity, which vary in units, scale, and noise characteristics. Furthermore, feature shapes such as resonance peak locations may be more informative than strict pointwise similarity. For this reason, we avoided including oversimplified scalar metrics in the Results section. However, to provide a basic quantitative summary, we calculated Spearman’s rank correlation coefficients (ρ) between the MAP-predicted spectra and the experimental mean data. This non-parametric measure reflects monotonic agreement across the frequency range, while remaining robust to nonlinear scaling.

The Spearman’s correlation coefficients between experimental data and model predictions ranged from 0.92 to 0.995, with *p*-values below 0.005 for stapes velocity, ear-canal input impedance, and absorbance. These strong correlations indicate that the FE model accurately captures experimental trends, even in the presence of variability. The coefficient of variation (CV) analysis revealed that the Young’s moduli of joints (CV = 0.0392) and eardrum (CV = 0.0283) had the highest certainty with minimal variability, while the Young’s modulus of ligaments (CV = 0.1197) showed the greatest uncertainty. The remaining parameters, with CVs ranging from 0.0545 to 0.0783, exhibited moderate variability, suggesting reliable parameter estimates and sufficient convergence of the inferred distributions.

A key advantage of SBI is its ability to integrate experimental noise and uncertainty during training. In our study, these uncertainties were quantified and incorporated using randomized STDs, which were applied to the simulation results. The trained NN effectively managed input noise up to 10 times the experimental STD, while maintaining accurate parameter probability distributions (Fig. 4). This result highlights the robustness of the SBI framework against elevated noise conditions. Increasing the noise level in training the SBI NN from 1 × to 10 × the experimental STD increased the uncertainty of parameter predictability, as reflected by an average 7 × rise in the CV. Despite this increase, the NN maintained high alignment with experimental data, with Spearman’s correlation decreasing only slightly from 0.96 to 0.94, indicating resilience to extreme noise.

Unlike traditional global search methods, which focus on finding a single best-fit parameter set through similarity measures like mean-squared error, the NN trained via SBI minimizes the Kullback–Leibler divergence (KL-D) between the true posterior and the NN’s approximate posterior^28^. This ensures that the returned distribution closely matches the Bayesian posterior, capturing the full range of plausible parameter values. This approach provides access to the entire posterior landscape, rather than a single solution, allowing for a more comprehensive characterization of model uncertainty.

### Model parameters and geometrical effects

As described in the Methods section, the model’s geometry was reconstructed from a micro-computed tomography (μCT) imaging scan of a temporal bone used for the physiological measurements. Soft tissues and their boundaries are often not well defined from μCT scans, necessitating manual segmentation of fine soft-tissue structures. This process introduces the potential for geometric inaccuracies, especially for small structures. The dimensions of small ME components, such as ligaments and eardrum thickness, may be comparable to voxel dimensions (e.g., 15.1 μm), leading to potential over- or under-estimation in segmented portions of the images.

Stiffness is proportional to both the thickness and Young’s modulus. In this study, SBI predicted the distribution of Young’s modulus for the stapedial annular ligament (SAL), which was in the kilopascal range and on the lower limit of the prior range (see Fig. 3)—a rarity for fibrous tissues^30^. One possible explanation is that the SAL thickness may have been overestimated during segmentation, which may have contributed to a lower inferred Young’s modulus to match the experimental data, reflecting the interaction of material properties and geometric structure on stiffness.

Conversely, the Young’s modulus of the joints exhibited the opposite trend. It appears that their geometrical dimensions were underestimated during segmentation, leading SBI to infer a higher Young’s modulus compared to the eardrum, even though fibrous tissues like the eardrum typically have a higher Young’s modulus. This underscores the role of geometry in shaping material parameter inference, highlighting the importance of accurate segmentation in computational modeling.

This study demonstrates parameter inference on a fixed anatomical model, where both the geometry and experimental data originate from the same temporal bone specimen. While this provides a strong proof of concept for amortized neural posterior estimation, future work will extend the framework to incorporate parameterized anatomical features. A practical method for geometry parameterization has already been developed and presented at the Mechanics of Hearing Conference (2024), enabling future models to account for both anatomical and material variability toward individualized, patient-specific diagnostics.

### General considerations

Applying SBI to tune models against experimental data requires balancing parameter count, simulator accuracy, and computational cost. This study simplified the model by omitting tissue viscoelasticity, thereby reducing the number of parameters. For instance, while the eardrum’s effective Young’s modulus varies with frequency^31^, it was treated as a constant. Similarly, cochlear load was simplified as a constant viscous force on the stapes footplate, which reduced computational expense^32^. All material parameters were assumed isotropic, though anisotropy, especially in the eardrum, affects stiffness^7^. Selected parameters were linked to ME pathologies; for instance, ossicular fixation correlates with ligament calcification and stiffness. Parameters like incus ligament stiffness were combined into one parameter due to their minimal impact on stapes velocity or ME metrics (Fig. 2). This approach reduces computational complexity while retaining essential model behavior. Understanding parameter influence typically requires prior knowledge or sensitivity analysis.

Experimental data variability arises from factors like tissue dehydration, instrument positioning, and background noise^33^, and simulations must replicate the experimental setup; inaccurate simulation conditions prevent convergence. This underscores the importance of aligning simulation conditions with experimental protocols to ensure convergence. An example of how data extraction location impacts outcomes is shown in Fig. S2.

In this study, a pre-simulated dataset of 10,000 was used to train the single-batch baseline NN, which completed training in under 30 min, even though simulations took several weeks. This approach supports rapid predictions for new experimental data without additional simulations and allows examination of internal NN parameters, including noise effects and density estimators. This rapid prediction capability significantly accelerates parameter inference for unseen data. However, for patient-specific models, iterative inference may be more computationally efficient, especially in cases with limited training data.

## Conclusions

This study demonstrates that SBI is an effective approach for tuning mechanistic models to accurately represent subject-specific clinical or experimental datasets. Unlike traditional sensitivity analysis, which requires subjective and labor-intensive tuning, SBI objectively infers model parameters, offering probability distributions for each parameter and revealing parameter interactions rather than isolated values. By capturing individual variability, SBI enables subject-specific computational models that can infer biological parameters within known normative ranges, supporting quantitative and objective assessments of normal versus pathological conditions.

While this study focuses on a human ME model, the approach is broadly applicable to other computational models, particularly in biological research. Using neural networks to infer probability distributions of model parameters, our approach offers a more comprehensive understanding of parameter interactions compared to single-value estimates. Importantly, by leveraging simulated data, the approach addresses the scarcity of clinical datasets, including those with confirmed pathological status. This makes the methodology generalizable to a broad range of biological models and well-suited for training inference neural networks.

This shift towards individualized modeling holds transformative potential for advancing objective diagnostics and personalized care. By better accounting for patient-specific variability and complexity in clinical data, SBI-based frameworks support personalized treatment planning and improved classification of normal versus pathological conditions. These capabilities lay essential groundwork for the development of patient-specific computational models, which are poised to have a significant impact on clinical diagnostics and intervention planning.

## Methods

This section outlines the pipeline for the simulation-based inference (SBI) methodology, followed by a detailed description of the experimental procedures used to obtain the reference empirical data for tuning the model. Next, we summarize the finite-element (FE) model development process and prior knowledge of the model parameters. Finally, we describe the procedure for running large-scale simulations, extracting data, and preparing it for training the SBI framework.

### SBI methodology

Figure 1 provides a schematic overview of the SBI methodology. After developing the simulator and selecting parameters of interest, plausible parameter ranges were defined based on data from the literature (Fig. 1A). For parameters lacking precise measurements, plausible ranges were derived from morphologically similar tissues, following established approaches^1,6^. Parameters were randomly sampled from these ranges and fed into the mechanistic model (Fig. 1B). Although parameter values were randomly sampled within prior ranges, any nonlinear interdependence among them was implicitly captured through the FE simulator and was reflected in the NN’s posterior distributions, especially the 2D histogram marginal plots (e.g., Fig. 2A). The mechanistic model generated simulated data based on the input parameter sets, and these simulation outcomes are collected (Fig. 1C) as described in Methods: Parameter selection, prior ranges, and model development. If necessary, dimensionality reduction can be applied to the simulation results to ensure efficient training of the neural network. As implemented here and described in another work^27,28^, dimensionality reduction was performed by passing the simulation outputs through an embedding network trained jointly with the density estimator.

Each input parameter set is paired with the corresponding simulation outcome and used to train a NN (Fig. 1D). The NN is trained to associate the input parameters with the output simulation results. Once trained, the NN can predict the posterior distribution of parameter values based on new input reference data (Fig. 1E). When the reference measurement data is used as input, the NN returns a probability distribution of the parameter estimates (posterior distribution, Fig. 1F), rather than a single fixed value. This approach provides a quantitative measure of the certainty associated with each parameter, allowing for a more comprehensive understanding of parameter interactions and uncertainty.

### Imaging and physiology measurements

The reference experimental measurements for this study were obtained from two independent sets of experiments. The first set involved wideband tympanometry, which measures the input impedance of the ME. This measurement allows for the calculation of ME absorbance, defined as the ratio of energy absorbed by the ME to the input acoustic energy. The second set of measurements was the stapes velocity, which characterizes transmission through the ME. The specific measurement procedures are described in prior work^34,35^. Seventeen sets of stapes velocity, wideband impedance, and absorbance spectra at ambient pressure were collected from test-retests on a single temporal bone (TB24). These measurement data were sourced from another project in our lab^36^.

Following the measurements, the temporal bone specimen (TB24) was transported to Boston University for μCT imaging. Imaging was performed using a Zeiss Xradia 520 Versa system with a resolution of 15.1 μm. The resulting image stack was imported into Simpleware (Version 2019; Synopsys, Inc., Sunnyvale, USA), where a combination of manual and automatic segmentation techniques was used to reconstruct the 3D geometry of the ME (Fig. 1B). The reconstructed model included the external canal (EC), eardrum, ossicles, ossicular joints, and suspensory ligaments and tendons. To reduce computational cost, the ME cavity was excluded from the model since it was open under the experimental condition. The load was applied as a prescribed volume velocity at the probe-EC interface, with simulations performed at 50 frequencies, logarithmically spaced between 250 and 8000 Hz.

To calculate impedance, the air pressure and volume velocity at the EC center was averaged on a 3 mm radius circular plane that was 3 mm from the probe-EC interface. This position mimics the distance between the speaker and microphone in the tympanometer probe used in the experiments^37^. The EC input impedance (Z_ec_) was calculated as the ratio of pressure to volume velocity^38^. The absorbance, defined as the ratio of the acoustic energy absorbed by the ME relative to the incident energy, was calculated from the impedance using the following equation:

1 $$Abs_{ec} = 1 - \left| {\frac{{{\raise0.7ex\hbox{${Z_{ec} }$} \!\mathord{\left/ {\vphantom {{Z_{ec} } {Z_{0} }}}\right.\kern-0pt} \!\lower0.7ex\hbox{${Z_{0} }$}} - 1}}{{{\raise0.7ex\hbox{${Z_{ec} }$} \!\mathord{\left/ {\vphantom {{Z_{ec} } {Z_{0} }}}\right.\kern-0pt} \!\lower0.7ex\hbox{${Z_{0} }$}} + 1}}} \right|^{2} ,$$

where $Z_{0} = {{\rho c} \mathord{\left/ {\vphantom {{\rho c} a}} \right. \kern-0pt} a}$ corresponds to the input impedance of an infinitely long tube. In this equation, ρ and c represent the density and speed of sound in air, respectively, and a is the lateral area of the EC.

The normalized stapes velocity (V_st_/P_ec_) was calculated as the ratio of the stapes velocity, obtained from the measured 3D stapes velocity projected in the piston-motion direction, to the pressure measured approximately 2 mm from the umbo. For both experimental measurements—wideband tympanometry and stapes velocity—the exact location of the measurement probe was not directly accessible, introducing a degree of uncertainty. To address this, a series of analyses were conducted to identify the optimal reference measurement location for the FE model. This was achieved through bulk simulations of the training dataset, as described in Supplementary Information (SI-2). We found that averaging pressure on a plane near the eardrum, parallel to the probe surface, yielded stapes velocity, EC impedance, and absorbance values consistent with experimental data. Without this alignment between simulation and experimental extraction locations, parameter variation alone failed to match the experimental data, preventing the SBI framework from converging on a posterior distribution of the model parameters.

### Parameter selection, prior ranges, and model development

We selected seven key ME parameters to tune our model: the Young’s moduli of the eardrum, mallear ligament, stapes annular ligament (SAL), ossicular joints, ME damping coefficient, and cochlear load. These parameters were selected due to their substantial influence on ME behavior and their known association with ME pathologies. For instance, stiffening of the mallear ligament is linked to ossicular fixation, while alterations in the stiffness of the SAL are linked to otosclerosis. Based on initial sensitivity analyses, we found that modeling each ligament and tendon separately would significantly increase simulation time without substantially improving spectral distinctions. Therefore, we reduced these into two aggregate parameters to maintain computational efficiency while preserving physiological relevance. Plausible ranges for these parameters were defined based on experimental data from the literature for normal MEs (Table 1). Prior bounds were also informed by experimental literature and selected to span approximately one order of magnitude above and below reported values. This choice reflects plausible biological variability while balancing computational feasibility and ensuring that meaningful posterior distributions can be learned within the prior space.

**Table 1:** Plausible ranges of the material properties of the FE model.

	Prior range	Reference(s)
Young’s modulus (MPa)		
Eardrum	1–20	^4,39^
Stapes annular ligament	0.005–1	^30,40–42^
Mallear ligament	1–10	^43,44^
Soft tissue (suspensory ligaments and tendons)	1–10	^10,18,43,45,46,46^
Joints	1–10	^47,48^
Damping (-)	0.05–0.2	^1,6,37,49^
Cochlear load (GΩ)	3–30	^50,51^

Parameters with less uncertainty and/or lower sensitivity—such as the Young’s modulus of ossicles (~ 14 GPa), the density of soft and hard tissues (1100 and 2100 kg/m^3^, respectively), and the Poisson’s ratio of soft and hard tissues (0.485 and 0.3, respectively)—were assigned fixed values from the literature^6,52^. These fixed parameters were not included as part of the SBI-tuned parameters.

The FE simulations were performed using COMSOL Multiphysics® software (Version 6.2) following the “Method” procedure outlined in^53^. Simulation data were directly exported using COMSOL’s batch export functionality^54^ to streamline large-scale simulations. We automated the randomized selection of parameter values within the prior ranges and imported each set into COMSOL. Parallel simulations were conducted using a hybrid shared and distributed memory approach^55^, with data processing handled by a MATLAB script (Version: 9.14.0 (R2023a)).

A total of 10,000 simulations were performed simultaneously on two computers. Each run simulated the system at 50 logarithmically spaced frequencies from 250 to 8000 Hz. The total computation time for all simulations was approximately four weeks, with each individual run requiring about 4.3 min to complete. This hybrid approach significantly reduced computation time and enabled the generation of a large dataset for SBI training.

### Training the inference neural network

We ran SBI using 10,000 parameter sets paired with corresponding FE model simulation outputs, which included the normalized stapes velocity V_st_/P_ec_, ME impedance Z_ec_ (both magnitude and phase), and absorbance Abs_ec_ magnitude. These outputs were concatenated into a single vector of 250 elements, representing the simulation responses across 50 logarithmically spaced frequencies for each measurement type.

To ensure robustness against experimental variability, Gaussian noise was added to this concatenated vector, with standard deviations calculated for each measurement type based on test–retest variability from experimental data. This approach enables the NN to learn from experimental variability, rather than overfitting to idealized data. Both the parameters and simulated traces were z-scored before being fed into the NN to standardize input scales.

The NN employed for SBI is a conditional density estimator, specifically a conditional neural spline flow (NSF), which employs a fully connected embedding network with three layers, each containing 50 hidden units (Durkan et al., 2020). This architecture facilitates flexible, non-linear approximations of posterior distributions, enabling the prediction of parameter posteriors given experimental data. The SBI toolkit is available on https://sbi.readthedocs.io/en/latest/.

## Supplementary Information

Supplementary Information.

## Data Availability

Data and Finite Element model (in COMSOL ver 6.2) for this study are available on the Harvard Dataverse site https://dataverse.harvard.edu/dataverse/Otobiomech, or from the corresponding author on request.
