# Supplementary material for: Simulation-based inference for subject-specific tuning of middle ear finite-element models towards personalized objective diagnosis
Source: Sci Rep. 2025 Nov 3;15:38364. doi: 10.1038/s41598-025-22164-2 (PMC12583476; doi:10.1038/s41598-025-22164-2)
Supplement: Supplementary file 1 — Supplementary Information. [file 41598_2025_22164_MOESM1_ESM.docx]

**Supporting Information**

Motallebzadeh et al. (DOI)

| 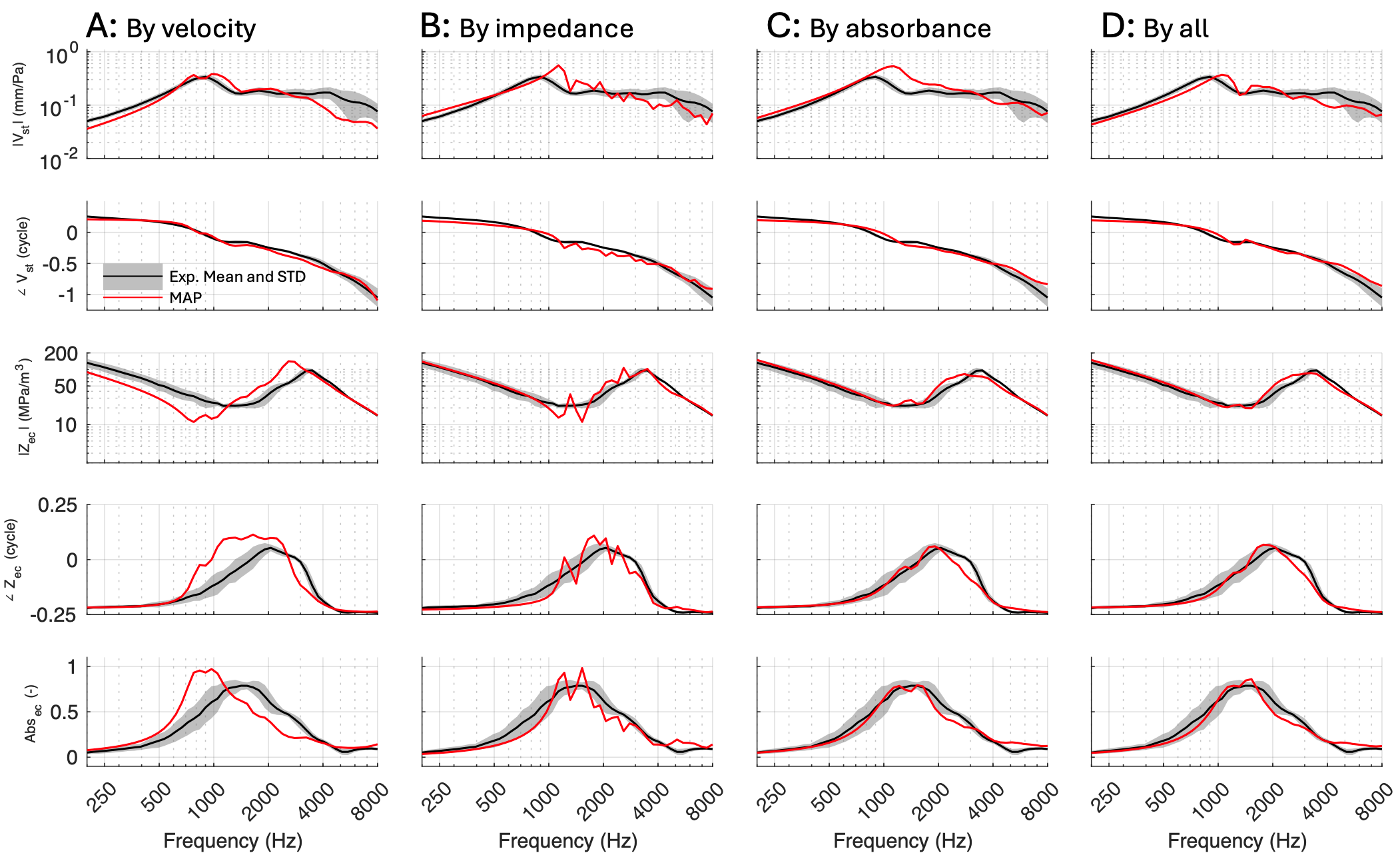 |
| --- |
| **Figure SI-1. Training Neural Networks with Individual or Combined Datasets.** Three neural networks (NN) were trained using simulation data from each dataset individually (A-Vst only, B-Zec only, and C-Abs only) and one NN (baseline NN in Fig. 3) was trained with all three datasets simultaneously (D). The maximum *a posteriori* (MAP) values of each NN were imported into the finite element simulator, and the resulting spectra are plotted against the experimental data. Each NN could reproduce the spectra corresponding to its training dataset but failed to accurately reproduce the spectra for the other datasets. In D, when all three datasets were used simultaneously to train the NN, the resultant MAP values could accurately reproduce all three objectives. |

| 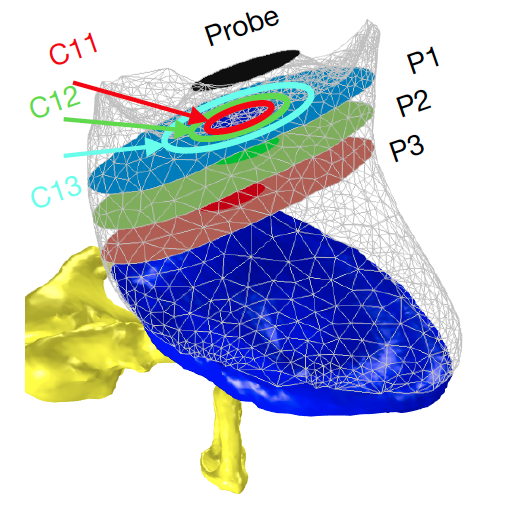 |
| --- |
|  |
| **Figure SI-2. Effect of data extraction location on the FE model.** To calculate ear canal input impedance and absorbance, acoustic pressure and volume velocity were measured in three cross-sections at planes 1 mm, 2 mm, and 3 mm (P1-P3, respectively) parallel to the probe tip surface (indicated by the black circle in A). At each plane, measurements were taken on three concentric circles with radii of 1 mm, 2 mm, and 3 mm, resulting in a total of nine measurement locations (CXY, where X is the plane number and Y is the radius of the circle). The cyan lines represent the experimental data (mean ± standard deviation), and the colored lines represent 100 samples of the training data. Only in C33 do the simulation data cover with the experimental data; in the other locations, systematic deviations occur (depicted by red circles) regardless of material parameters. |
